# Multianticipation for string stable Adaptive Cruise Control and increased motorway capacity without vehicle-to-vehicle communication

**DOI:** 10.1016/j.trc.2022.103687

**Published:** 2022-07

**Authors:** Riccardo Donà, Konstantinos Mattas, Yinglong He, Giovanni Albano, Biagio Ciuffo

**Affiliations:** a Uni Systems Italy, Milan, Italy; b European Commission Joint Research Centre, Ispra (VA), Italy; c University of Cambridge, Cambridge CB3 0HA, UK; d Seidor Italia Srl, Milan, Italy

**Keywords:** Adaptive cruise control, Car-following, Multianticipation, String stability, Traffic dynamics, Microsimulation

## Abstract

Adaptive Cruise Control (ACC) systems have been expected to solve many problems of motorway traffic. Now that they are widespread, it is observed that the majority of existing systems are string unstable. Therefore, small perturbations in the speed profile of a vehicle are amplified for the vehicles following upstream, with negative impacts on traffic flow, fuel consumption, and safety. Increased headway settings provide more stable flow but at the same time it deteriorates the capacity. Substantial research has been carried out in the past decade on utilizing connectivity to overcome this trade-off. However, such connectivity solutions have to overcome several obstacles before deployment and there is the concrete risk that motorway traffic flow will considerably deteriorate in the meanwhile. As an alternative solution, the paper explores multianticipation without inter-vehicle communication, taking advantage of the recent advancements in the field of RADAR sensing. An analytical study is carried out, based on the most widely used model and parameter settings used to simulate currently available commercial ACC systems, comparing the transfer functions and step responses for the nominal and the multianticipative formulations. Then, a microsimulation framework is employed to validate our claim on different speed profiles. Analytical results demonstrate that multianticipation enhances stability without impacting traffic flow. On the contrary, the simulation study indicates that the multianticipative-ACC can produce higher road capacity even in the presence of external disturbances and for a wide range of calibrated parameters. Finally, optimality conditions for the tuning of the headway policy are derived from a Pareto optimization.

## Highlights

• Commercial ACC systems have a string-unstable behavior.
• String-stable ACCs rely on the use of communication which is not currently available.
• ACC can still be string stable by taking advantage of RADAR sensing characteristics.
• Monitoring two vehicles downstream is sufficient to ensure string-stability in a wide range of scenarios.
• RADAR based multianticipation can provide a plug-and-play solution to achieve efficient traffic.

## 1 Introduction

The development of several Advanced Driver Assistance Systems (ADAS) is paving the road to full automation of highway driving. Systems such as the Adaptive Cruise Control (ACC), which regulates the velocity of a vehicle with respect to a leader vehicle, are reshaping the way drivers approach the driving task.

There have been high hopes in the research community on the potential benefits that the ACC could bring to road traffic dynamics (Kesting et al., 2008, van Arem et al., 2006). A consistent research effort was pursued to design optimal ACC controllers that would annihilate phantom traffic jams. Notable examples from this line of research can be found, for instance, in (Magdici and Althoff, 2017, Nilsson et al., 2016, Wang, 2018). Nonetheless, a parallel line of research has also been established which aims at verifying the actual behavior of commercial ACC systems. It was found that the assumptions used in the theoretical scientific contributions (*i.e.,* small time headway, negligible response time, and string stability) were not always backed by the experimental evidence. Furthermore, obtaining insights into the ACC operation has turned out to be a difficult task due to the proprietary nature of the control strategies (He et al., 2019).

Within the second line of research, a pioneering work has been carried out by Shladover et al. (Shladover et al., 2012), using real data both for the ACC driving behavior and the people’s preferences on the desired headway setting. It was shown that the time-gaps used were not significantly shorter than those used by human drivers. Therefore, capacity would not increase significantly. Shladover et al. (Shladover et al., 2012) have also mentioned that the response time of current commercial ACC systems may be non-negligible, and their behavior may be string unstable without presenting an extensive investigation of those attributes.

Since then, experiments on commercial ACC vehicles have become easier to carry out, with the ACC systems being widespread and not just available in high-end vehicles, and with the availability of more affordable tools to obtain accurate trajectory data. The reaction time of such systems has been observed to be similar to that of a human driver (Makridis et al., 2018, Makridis et al., 2020). Furthermore, different research teams, working in parallel, have produced data showing that commercial ACC systems are indeed *string unstable* (Makridis et al., 2020, Gunter et al., 2021, Shi and Li, 2021, Ciuffo et al.).

In a string stable platoon, a perturbation would decrease in magnitude while traveling upstream (Wilson and Ward, 2011, Wilson, 2008). For string unstable platoons, even a small perturbation would be amplified upstream, often leading to stop-and-go waves in the case of long vehicle platoons. Such traffic oscillations have long been recognized as an important problem of road traffic systems (Herman et al., 1959, Edie, 1961), inducing congestion and more frequent safety–critical situations, and worsening environmental impacts (Zheng et al., 2010, Bilbao-Ubillos, 2008). With more research dedicated to the issue of string stability and automated driving, a very troublesome trade-off has become apparent between string stability and capacity. Larger time-gap settings tend to lead to more string stable platoons (Shi and Li, 2021, Makridis et al., 2021, Zhou et al., 2104). However, vehicles maintaining large time-gaps would negatively affect the capacity, decreasing the potential traffic flow (Mahmassani, 2016).

Vehicle-to-Vehicle (V2V) communication has been proposed as the predominant solution to address the mentioned trade-off with the Cooperative Adaptive Cruise Control (CACC) systems. The potential impact of CACC on traffic flow, energy consumption, and time delays has been evaluated by simulation experiments (Mattas et al., 2018) and experimental validation (Ploeg et al., 2011). In particular, in (Ploeg et al., 2011), a topology (two predecessors leader following) which closely resembles our work was adopted. However, the market penetration of connectivity is still negligible and plagued by several challenges, including concerns with data privacy, infrastructure availability, lack of communication standardization, and cybersecurity threats (Wang et al., 2018, van der Heijden et al., 2017, Mahdinia et al., 2020). Such systems may become vulnerable to cyber-attacks that would jeopardize the potential benefits of safety and traffic flow stability (Khattak et al., 2021). Proper methods to establish resilience to cyber-attacks are being researched (Zou et al., 2020). Furthermore, legal issues have to be resolved regarding the data privacy and data ownership of the information that will be exchanged (Suo et al., 2021).

A possible alternative could be the use of multianticipation, *i.e.*, reacting to the state not only of the leader vehicle but of multiple vehicles ahead. It has been observed that multianticipation could stabilize the traffic flow (Chen et al., 2009) and that human drivers often use information from vehicles downstream (Lu et al., 2015).

Building up on the valuable contribution of multianticipation, this work explores a solution to enhance ACCs’ functioning by taking advantage of the distance and velocity information of the second leader ahead in the platoon. Thus, the car-following policy constitutes a realization of the multianticipation mechanism described in (Chen et al., 2009), albeit restricted by relying on two leaders only. The current work stands apart from state-of-the-art contributions dealing with CACC by freeing the vehicles from needing communications modules to share information, relying instead on onboard sensors only. In particular, we devise the exploitation of multipath reflections in RADAR (Holder et al., 2018) to detect obstacles that are not directly in the line-of-sight of the sensor. Recent studies have also highlighted how RADARs can even perceive around corners (Scheiner et al., Thai et al., 2017) utilizing a similar principle. Such a technology, to the best of the authors’ knowledge, is exploited by Tesla since the release of AutoPilot v8.0 commercial software (Tesla AutoPilot v8.0, 2016) for a purpose comparable to the scope of the current work.

**Fig. 1:**
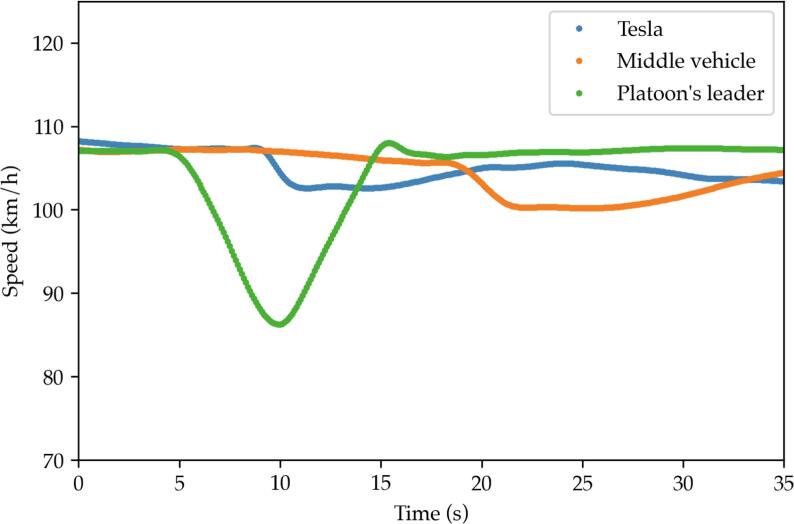
Experimental verification of Tesla's multianticipation, second leader braking scenario.

An experimental testing campaign carried out by the authors has corroborated the existence of such multianticipation mechanism for a Tesla Model 3 MY2021 sold in the European market^1^. The details of the experimental verification go beyond the scope of the current article and are discussed in an upcoming manuscript (Donà et al., 2021). Nonetheless; the authors can report that multianticipation did prove to be mainly tuned for safety by managing to provide better safety metrics than a traditional ACC system for a range of car-following and cut-in traffic scenarios. For example, Fig. 1 depicts the recorded velocity profiles of the three vehicles making up the platoon during a car-following experiment where the Tesla was the last vehicle. The platoon is traveling at 110 km/h when a perturbation is induced by the ‘Platoon’s leader’. It is easily noticeable that, despite the very late reaction of the in-between vehicle, the Tesla *anticipated* the braking and reacted to the perturbation induced by its second leader, which was not in its direct line of sight.

Currently, a theoretical assessment of the expected traffic flow and safety improvements arising from the adoption of such a “limited” multianticipation mechanism is missing in the literature, which is precisely the scientific contribution this paper aims to address. The relevance of the work is further motivated by the availability on the market, and, consequently, on public roads, of vehicles equipped which such a technology.

The paper’s goal is thus to examine how effectively the second leader position and velocity information could be exploited to enhance current ACC systems. To this end, we approximate the functioning of the ACC car-following behavior exploiting a linear model that is used in the literature by the vast majority of studies concerning commercial ACCs system (Shladover et al., 2012, Gunter et al., 2021, Shi and Li, 2021). Despite there might be more performing car-following models, we deliberately decided to adopt the linear modeling framework given the supporting evidence suggesting its capability of replicating the real-world behavior observed for such systems, and the stability analysis that the linear formulation can afford. Moreover, given our proposal for multianticipation is largely based on ACC-derived technology, the same set of parameters as the ones obtained from experimental ACC calibrations in the scientific literature is also used for the multianticipative solution. This choice enables accounting for technological limitations behind the tuning of ACC systems for the sake of a fair comparison between the two solutions. Any performance difference can thus be ascribed to the efficient use of the second leader information and not to the specific tuning of the controller. We eventually give a quantitative measure of the increase in the potential of addressing the traffic flow and string stability trade-off for a wide range of controllers’ realizations.

### 1.1 Literature review

With the advent of vehicle automation, the interest in researching string stability has increased substantially. The string stability of heterogeneous traffic flow has been investigated (Montanino and Punzo, 2021). In different experiments, several research teams have found current commercial ACC systems to be string unstable (Makridis et al., 2020, Gunter et al., 2021, Shi and Li, 2021). This could increase the congestion problems, and heavily influence the energy impacts of ACC, according to an analysis of experimental data (He et al., 2020). Overall, in contrast to earlier studies, vehicle automation without connectivity is no longer expected to improve the condition of the traffic networks significantly (Shladover et al., 2012, Mattas et al., 2018, Calvert et al., 2017, Shang and Stern, 2021).

Introducing the influence of multiple leaders in driving behavior is not a novelty, and there is indeed considerable literature supporting the advantages of multianticipation. Early studies on multianticipation date back to the late ‘60 s, with Bexelius (Bexelius, 1968) extending the existing car-following model to account for additional vehicles. Bexelius’ seminal work contributed to instantiate a research line still active nowadays (Farhi et al., 2012, Lenz et al., 1999, Ossen and Hoogendoorn, 2006) in modeling humans’ car-following behavior. Microscopic simulation models, including multianticipation, have shown to be suitable to simulate the human driver behavior based on data collected from a helicopter (Hoogendoorn et al., 2006). Nirmale et al. (Nirmale et al., 2021) used trajectory data from Chennai, India, to show that human drivers react to many vehicles around and downstream. Multianticipation has proven to be a stabilizing factor for traffic flow (Monteil et al., 2014, Ngoduy, 2015, Sun et al., 2018). It has been shown that multianticipation can also stabilize platoons of vehicles that operate with large reaction times (Treiber et al., 2007). However, those works focus on human drivers and the accurate reproduction of their behavior, not on Automated Driving Systems (ADS) development.

Multiple leaders’ anticipation is also a consolidated topic within the community dealing with CAVs. In particular, many works exploit inter-vehicles connectivity to enable platoons to share vehicles’ information. Solutions such as the CACC allow better string stability properties and reduced headway to make full exploitation of the road capacity (Chen et al., 2019, Dey et al., 2016). Information about preceding vehicles can be used to make predictions, aiming for higher safety and stabilized traffic flow (Sainct, 2020). Platoons can be robust, even maintaining a decentralized cooperation strategy (Zhou and Ahn, 2019). The influence of multianticipation has been considered even in macroscopic models derived from microsimulation models (Nateeboon et al., 2019). Interestingly enough, while the effects of multianticipation have been investigated for human drivers, and multianticipation is used in the design of connected driving behaviors, to the best of the authors’ knowledge, it has not been proposed as a suitable solution for non-cooperative automated driving, such as ACC systems.

Our scientific contribution hence stands apart from solutions based on communication by relying uniquely on the RADAR sensor. On one side, the RADAR can only provide the second leader’s position and velocity thanks to the multipath propagation effect. On the other hand, this technology has already hit the market and represents a viable option to increase ACCs’ safety and performance today. Therefore, its applicability in the real-world could be faster than any other solution to improve ACCs’ operations. A simulation implementation of the complex mechanisms underlying the RADAR functioning goes beyond what this paper can deliver for the sake of simulating car-following scenarios. Nonetheless, precautionary hypotheses are adopted to account for the potential inaccuracies that such a solution might be subjected to.

The present paper is organized as follows. In section 2, the model used for the analytical analysis is first derived. Some important properties directly stemming from the updated formulation of the control law are then presented in Section 3. Section 4 focuses on the derivation of macro-traffic metrics via exploiting the additional damping potential for the sake of maximizing flow. The analysis concludes by presenting the simulation environment and the simulation results in section 5. Conclusions and broader implications are eventually discussed in section 6.

## 2 Model derivation

This section is concerned with defining a suitable model for the ACC realization, which will be then enhanced to accommodate the additional second leader sensor information. The ACC’s proposed model is the well-known linear controller (He et al., 2019) and is briefly delineated in 2.1 ACC model. Afterward, in Section 2.2, the augmented control law is outlined together with the introduction of the second leader’s relative importance to the actual control effort.

The parameters’ selection for the models here described (both the ACC and the M−ACC, since no calibrated parameters exist in the literature for vehicles potentially equipped with M−ACC) stems from (Gunter et al., 2021, Shi and Li, 2021) together with some calibrations we performed based on the trajectories collected in the OpenACC dataset (He et al., 2107). The RADAR-specific noise and delay figures are based on the work presented in (Hasch et al., 2012). Throughout the current Section, a reference controller is considered for the study whose parameters are reported in Table 1 (approximately corresponding the “mean” controller resulting by averaging each parameter in Table 2). On the contrary, the effect of multianticipation over the interval of gains and delays reported in the literature is systematically analyzed in Section 3.3 and Section 4 for the parameters in Table 2.

**Table 1:** Parameters used to compute the transfer functions and step responses.

Parameter	$k_{p}$	$k_{d}$	$t_{g}$	τ	$T_{1}$, $T_{2}$	η
Value	0.13 (1/s^2^)	0.4 (1/s)	1.75 (s)	0.1 (s)	0.75 (s)	2.0 (m)

**Table 2:** Parameters ranges used in the micro-simulation environment.

Parameter	Lower bound	Upper bound	Unit
$k_{p}$	0.03	0.25	(1/s^2^)
$k_{d}$	0.25	0.70	(1/s)
$t_{g}$	1.20	2.50	(s)
τ	0.10	0.50	(1/s)
$T_{1}$	0.50	1.50	(s)
$T_{2}$	$T_{1}$ + 0.00	$T_{1}$ + 0.50	(s)

### 2.1 ACC model

The work builds up on the widely adopted kinematic linear model (PD controller), which has been demonstrated to be an excellent candidate for modeling the ACC behavior (He et al., 2019). The plant behavior is approximated by the third-order kinematic model

(1) $$\left\{\begin{array}{c} \dot{s}\left(t\right)=v_{L}\left(t\right)-v\left(t\right)\\ \dot{v}\left(t\right)=a\left(t\right)\\ \dot{a}\left(t\right)=\frac{u\left(t\right)-a\left(t\right)}{\tau }, \end{array}\right)$$

where *s(t)* is the distance between the leader and the ego follower projected positions along the curvilinear abscissa, $v_{L}$*(t)* is the leader’s speed, *v(t)* the ego’s speed, *a(t)* the chassis acceleration of the ego vehicle, *u(t)* the commanded acceleration and τ the actuation’s time constant. Notice that, as an alternative formulation, the absolute coordinates $x_{L}\left(t\right)-x\left(t\right)$ could have been used equivalently to *s(t)*. The manipulated input *u(t)* affects the jerk of the system via a first-order dynamics which is representative of the driveline and engine response time. The model in (1) allows accounting for the actuation lag, which has been reported to play a significant role while studying the string stability properties of a platoon (Xiao and Gao, 2011, Wang et al., 2018).

In order to provide the system in (1) with car-following capabilities, a spacing policy has to be introduced. In particular, we selected the widely adopted constant time headway (CTH) control policy

(2) $$u\left(t\right)=k_{d}\left(v_{L}\left(t-T\right)-v\left(t\right)\right)+k_{p}\left(s\left(t-T\right)-t_{g}v\left(t\right)-\eta \right).$$

In (2), *t_g_* is the desired time-gap (bumper to bumper time-difference), η the standstill spacing, and *T* the estimation delay. Applying the control law (2) to the ACC system in (1) the state-space realization

(3) $$\left[\begin{array}{c} \dot{s}\left(t\right)\\ \dot{v}\left(t\right)\\ \dot{a}\left(t\right) \end{array}\right]=\left[\begin{array}{ccc} 0 & -1 & 0\\ 0 & 0 & 1\\ \frac{k_{p}\lambda_{T}}{\tau } & -\frac{k_{d}+{k_{p}t}_{g}}{\tau } & -\frac{1}{\tau } \end{array}\right]\left[\begin{array}{c} s(t)\\ v(t)\\ a(t) \end{array}\right]+\left[\begin{array}{c} 1\\ 0\\ \frac{k_{d}\lambda_{T}}{\tau } \end{array}\right]v_{L}\left(t\right)$$

can be drawn for the ACC realization, where the $\lambda_{T}$ operator introduces the finite time estimation delay λ(t-T). Ultimately, in (3), every parameter is supported by physical meaning, which enables robust string stability analysis using system dynamics tools for a wide range of controller realizations.

### 2.2 M−ACC model

The control law in (2) can be straightforwardly extended to accommodate the extra terms related to the state of the second leader as in

$$u\left(t\right)=k_{d}\left(v_{L1}\left(t-T_{1}\right)-v\left(t\right)+w_{L2}\left(v_{L2}\left(t-T_{2}\right)-v\left(t\right)\right)\right)$$

(4) $$+k_{p}\left(s_{1}\left(t-T_{1}\right)-t_{g}v\left(t\right)-\eta +w_{L2}\left(s_{2}\left(t-T_{2}\right)-2t_{g}v\left(t\right)-\eta \right)\right),$$

where *s_2_(t)* and $v_{L2}\left(t\right)$ are respectively the distance and the velocity of the second leader. Notice that, differently from anticipative CACC alternatives, the proposed solution does not rely on the leaders’ acceleration information. Conversely, all the input quantities to (4) can be derived from RADAR’s generated evidence. Moreover, the bounds assumed for the noise and delays in Table 2 are the typical figures a RADAR system can deliver which emphasizes the diversity of our approach with respect to CACC studies.

**Fig. 2:**
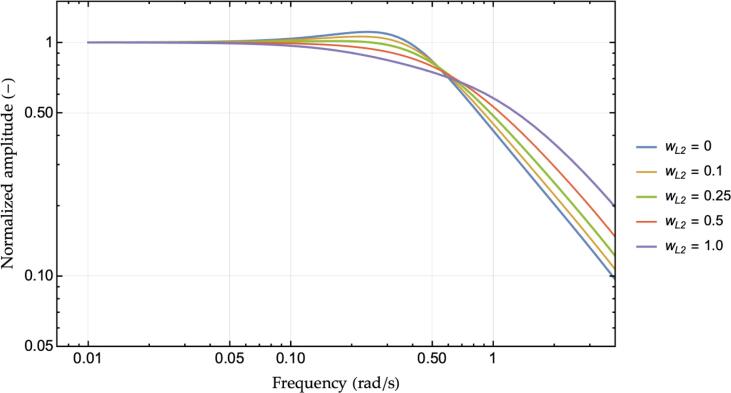
Transfer function chart from first leader’s speed to ego vehicle’s speed, ACC vs. M−ACC for several values of $w_{L2}$.

In (4), the design parameter “$w_{L2}$” controls how much the designer trusts the second leader’s distance and velocity measurements. Setting $w_{L2}$ to zero would indeed return the same control law as the original ACC in (2), whereas regulating $w_{L2}$ to one yields a controller which reacts to the second leader in the same way as for the first. Given no direct practical estimation of $w_{L2}$ is available, we cannot give an empirical bound to the coefficient. In the remaining of the paper, $w_{L2}$ will be kept fixed at 0.5 with except for the string stability analyses in Fig. 2 and Fig. 4, where the coefficient is iterated to illustrate its significance in stabilizing the platoon. The definition of optimality criteria for $w_{L2}$ is outside the scope of the current work and is demanded to future activities once an experimental characterization of the proprietary technology is available. Nonetheless, in (4), the relative importance of the second leader can be modulated using other methods apart from balancing $w_{L2}$. For instance, the second leader spacing error could be clipped in order not to affect safety when traveling under a short headway setting while preserving the reactivity of the multianticipation. However, in our contribution, we intentionally decided not to introduce non-linear components to emphasize the comparison with (2) and taking advantage of linear system stability analysis techniques. Moreover, being ACC and M−ACC Level 1 or 2 automation technology (SAE On-Road Automated Vehicle Standards Committee, 2021), a safety driver shall always be present and ready to take over in emergency conditions.

Concerning the equilibrium distances, while the ACC under the CTH policy has an equilibrium spacing function which only depends on the travelling velocity, once the velocity difference with respect to the leader is annihilated, the M−ACC shows a *locus* of equilibria points due to the extra input quantities affecting the control law. In particular, under the assumption of equal velocity with respect to the leaders, the equilibrium region is given by a plane in the $s_{1}\left(t\right),s_{2}\left(t\right),v\left(t\right)$ space. Conversely, the M−ACC equipped vehicle will tend to use larger headway than the equivalent ACC when the first leader is tailgating its leader. On the other side, the anticipative system will use shorter than nominal headway in case the first leader is using large time-gaps. Such a setup makes comparing the capacity beforehand quite cumbersome. Hence, we will study if any capacity reduction or enhancement occurs via simulation to better explore the behavior under randomized headway configurations for the ego-vehicle/leaders.

Notice that the gains of the controller remain unchanged upon the introduction of the second leader information. In addition, the control law (4) is given a versatile formulation by allowing, in principle, the estimation delay of the second leader $T_{2}$ to be different from the first leader’s delay $T_{1}$.

Similarly to (3), the control law in (4) gives rise to a state-space formulation which we refer to as the “M−ACC”. The model in (4) can also be seen as a *Generalized Helly* (Ossen and Hoogendoorn, 2006) for a two leaders case where a design parameter modulates the relative importance of the second leader.

## 3 Analytical assessment

This section is devoted to the evaluation of the performance of the M-ACC system with respect to the original ACC formulation. The assessment is here carried out by means of an analytical study which is supported by transfer functions and step responses computations. The outputs are computed for both a nominal reference plant and for a “perturbed” model, where the estimation delay is manipulated to inspect the robustness. Finally, the impact of the control law (4) over a large set of controllers’ gains is discussed.

### 3.1 M−ACC Nominal performance

This set of analyses is concerned with computing the transfer function from the leader vehicle ($V_{\text{leader}\;}\left(s\right)$) to the ego vehicle ($V_{\text{ego}\;}\left(s\right)$) and with the evaluation of the unitary step response for the systems ACC and M−ACC. In particular, the mentioned transfer function magnitude

(5) $$\|\frac{V_{\text{ego}\;}\left(s\right)}{V_{\text{leader}\;}\left(s\right)}\|,$$

is also known as the “complementary sensitivity criterion” (Ploeg et al., 2014), and it is a widespread metric for assessing the string stability of a leader/follower pair.

The parameters used throughout this section are reported in Table 1, and they are representative of the average ACC controller used in the simulation section as from the parameters’ bounds in Table 2. $w_{L2}$ was kept, instead, as a varying parameter. The chosen gains do not stem from any optimization of the car-following capabilities, neither for the ACC nor the M−ACC controllers. Nonetheless, they afford grasping the transition from the string unstable to the string stable region as $w_{L2}$ is gradually increased. The effectiveness of applying multianticipation to other controller’s realizations is discussed in the continuation of the paper.

Fig. 2 displays the transfer function magnitude (5) for parameters in Table 1 for a set of $w_{L2}$ ranging from 0 (ACC functioning, blue line) to 1.0 (fully multi-anticipative behavior, violet line). It is clear how the M−ACC logic provides the system with a significant damping boost as the ACC solution exhibits a quite significant resonance peak which is progressively reduced for increasing values of $w_{L2}$.

**Fig. 3:**
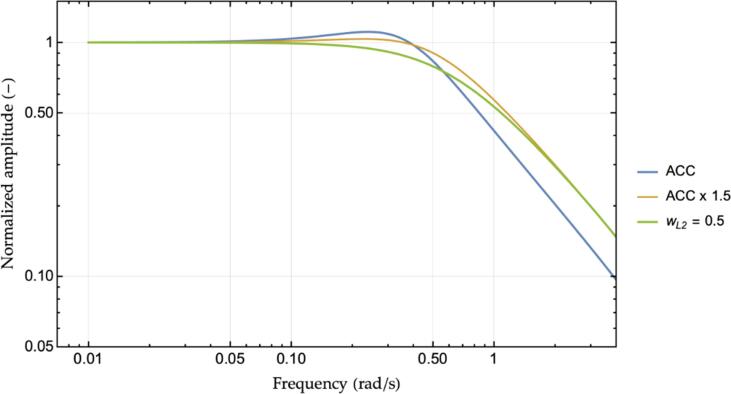
Reference ACC controller transfer function vs. re-scaled ACC controller and M−ACC enhanced version.

Notice that the introduction of multianticipation is not equivalent to the re-scaling of the original ACC’s control law by a factor equals to ${1+w}_{L2}$ to artificially increasing the gain. Such a case is shown in Fig. 3, where the reference ACC controller, as of Table 1, is compared against its re-scaled (times ${1+w}_{L2}$) gains’ parametrization and the M−ACC enhancement using $w_{L2}=0.5$. Indeed, increasing the ACC’s gains provides better stability with respect to the original formulation. However, the extra damping of the M−ACC realization proves to derive from the change in the dynamical behavior of the system, namely, the additional damping terms in the dynamical matrix of the system arising from the revised closed-loop formulation, rather than from the larger absolute magnitude of the control law that might occur due to the additional input.

**Fig. 4:**
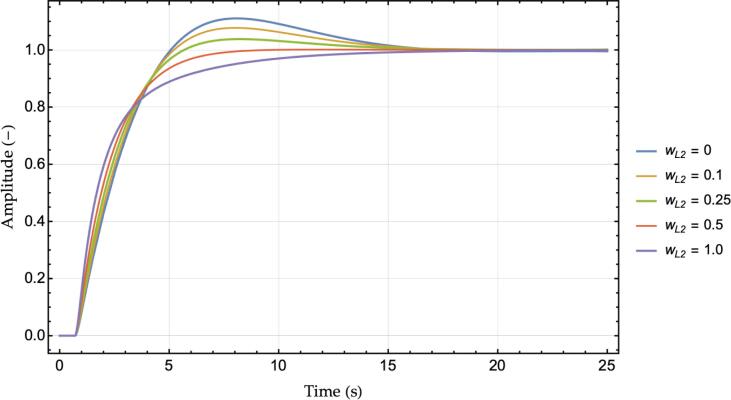
Step responses chart, ACC vs. M−ACC for several values of $w_{L2}$.

Fig. 4 depicts instead the step response in terms of the produced velocity of the ego vehicle for a unit step change in the leader velocity. Similarly to Fig. 2, the overshoot is significantly damped for increasing the value of $w_{L2}$. The step response also highlights the delay in the reaction to the leader speed change as the system's response starts only after 0.75 *s*. The last consideration is related to the chassis acceleration: the M−ACC appears to use more instantaneous acceleration as the slope of the violet line is noticeably larger than the ACC case in the transient phase of Fig. 4. However, it will become apparent in the simulation section that the actual RMS value of the acceleration of an M−ACC in a real-world scenario is lower than the dual ACC due to the additional stability that characterizes its functioning.

### 3.2 M−ACC Robustness

**Fig. 5:**
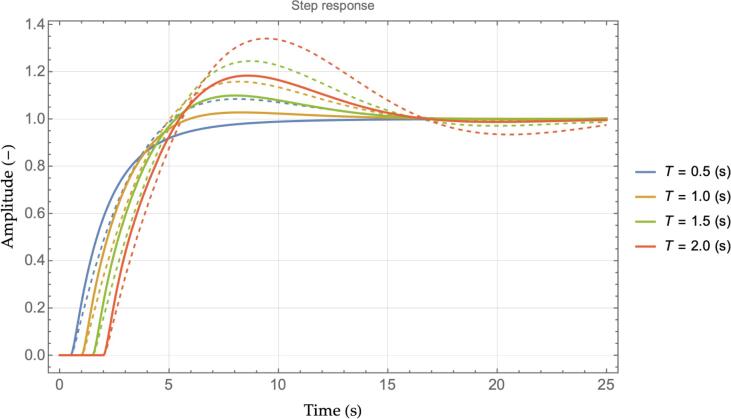
Step responses for different estimation delays, ACC (dashed lines) vs. M−ACC (solid lines), $w_{L2}$= 0.5.

The formulation proposed relies on a set of modeling assumptions that, in practice, may not be fully satisfied at any time. Firstly, every measurement is affected by noise. Interestingly, ensuring compliance with the complementary sensitivity criterion (5) also provides the system with less susceptibility to noise as the system will not amplify the absolute value of any input signal being the magnitude of the transfer function always ≤1. Secondly, the estimation delay of the leader’s state may not be constant. The impact of different estimation delays is investigated in Fig. 5 by means of comparing the step responses of the M−ACC and ACC systems for different values of *T,* while the other parameters derive from Table 1. As expected, increasing *T* leads to larger overshoots for both the ACC (dashed lines) and the M−ACC (solid lines) cases. Remarkably, however, the M−ACC manages to return better performance than the ACC for the full range of considered delays suggesting a desirable robustness property.

**Fig. 6:**
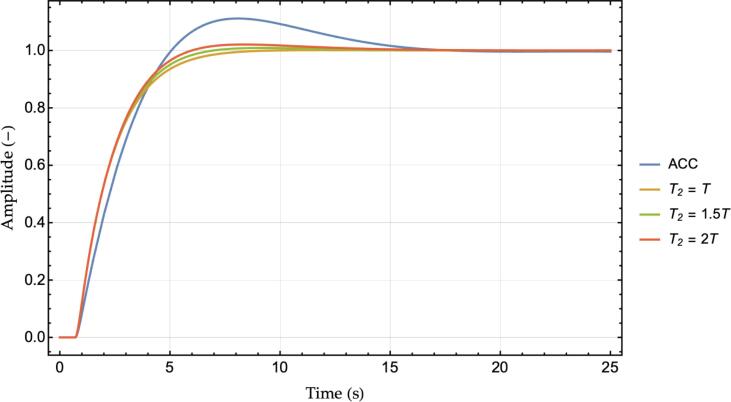
Step responses, effect of additional delay $T_{2}$, $w_{L2}$= 0.5.

Lastly, the estimation of the second leader’s state might require extra time with respect to the perception of the first leader, hence $T_{2}\geq T_{1}$. Fig. 6 investigates the impact of different $T_{2}$ by keeping fixed any other parameter according to Table 1. As it can be noticed, even in the case of twice the estimation time, the M−ACC provides substantial performance gain over the traditional ACC solution.

### 3.3 M−ACC generalization

Nonetheless, all the considerations reported up to now are limited to the specific set of parameters used to compute the transfer functions and step response in Table 1. One may wish to systematically establish the performance of the M−ACC control law for a broader range of calibrated parameters to grasp the actual potential of multianticipation.

**Fig. 7:**
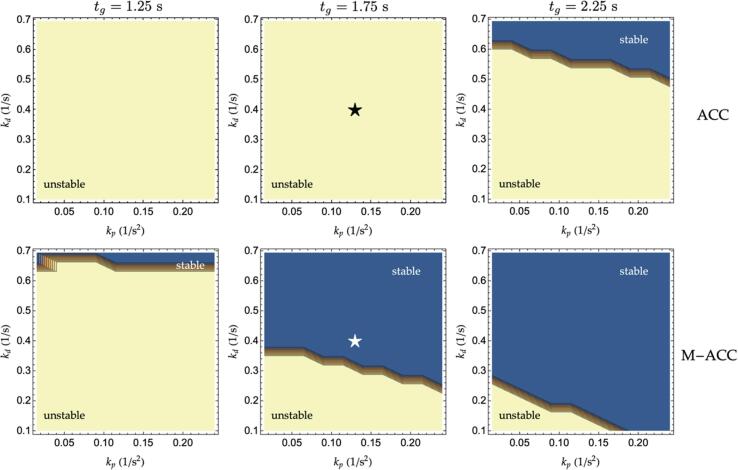
Stability regions of ACC vs. M−ACC, $w_{L2}$= 0.5, τ=0.1s, T = 0.75 s.

This paragraph demonstrates such a generalization property by establishing the string stable regions in the $k_{p}-k_{d}$ plane for a set of candidate time-headways. The procedure involves computing, for each set of considered $k_{p}$*,* $k_{d}$ and for a list of selected time headways, the maximum absolute value of (5). The obtained results are graphically reported in Fig. 7 for both the ACC (top charts), and the M−ACC (bottom charts) control laws. The additional string stability potential can be easily grasped by noticing the wider blue areas (stability regions) for the M−ACC with respect to ACC. Interestingly, the impact of headway on the M−ACC is analogous to the ACC, *i.e.*, an increase in the headway is associated with a larger stability region.

Notice also how the reference controller (indicated by the star symbol in Fig. 7, parameters in Table 1) shifts from the unstable area to the stability region coherently with the transfer function (Fig. 2) and step response (Fig. 4) computations after the introduction of multianticipation with 50% of trust of the second leader. It is important to note that the average time-gap human drivers maintain has been estimated to be 1.4 *s* in the literature (Xiao et al., 2018), and ADS would have to maintain shorter gaps to improve the capacity. According to Fig. 7, string stable platoons with reduced headway with respect to the average human are not possible without multianticipation for the control gains taken into consideration.

**Fig. 8:**
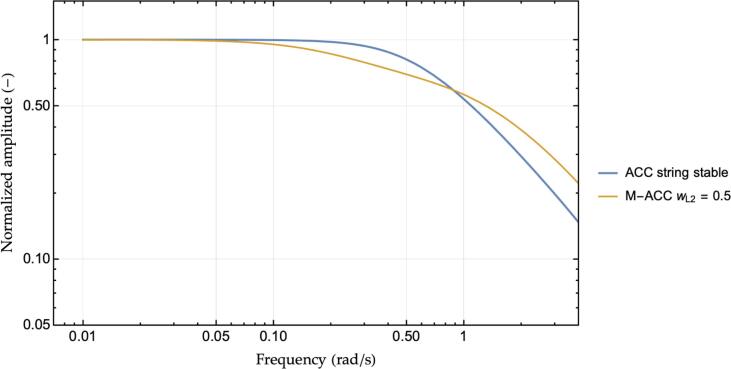
String stable ACC and its M−ACC extension transfer functions.

Concerning instead the cases where the ACC controller is string stable, their M−ACC generalizations maintain the string stability property while, at the same time, increasing the attenuation of the higher frequency content. Considering, for instance, a string stable ACC controller featuring $k_{p}=0.2,k_{d}=0.6$ and $t_{g}=2.25$ s, its M−ACC extension’s magnitude transfer function is shown in Fig. 8 together with the original ACC’s output.

Such additional damping visible in Fig. 8, despite not contributing to the string stability argument already satisfied by the original ACC, might enhance heterogenous platoons’ operation where also non-string stable vehicles could participate.

## 4 Macro-traffic metrics

From the previous section, updating the control law (2), (3), (4) resulted in a *damping* enhancement that boosts the M−ACC string stability property. However, the potential benefit gained can be exploited for the sake of maximizing flow rather than string stability (or any trade-off solution in between). In particular, the M−ACC controller could be allowed to travel with a reduced headway while providing at the same time the same string stability property of the original ACC. This section proposes an equivalence criterion based on the transfer function amplitude that allows translating the beneficial effect of multianticipation into flow improvement.

### 4.1 Equivalent transfer function amplitude

In order to quantitatively assess the effect of the proposed multianticipation control law on flow increase, the transfer function modulus in (5) here exploited in an iterative procedure. Firstly the maximum amplitude of the transfer function is evaluated for the nominal ACC plant, then the M−ACC is derived via updating (2), (3), (4), and the corresponding transfer function is computed. Lastly, the time-gap for the M−ACC is iteratively reduced until the maximum amplitude of the M−ACC matches the one of the ACC. The obtained time-gap represents *de facto* an equivalence measure to translate the additional damping potential into flow increase. Notice that the application of the M−ACC control law does not yield any flow increase *per se* at the equilibrium conditions unless the headway is shortened using an equivalence criterion such as the iterative procedure explained.

**Fig. 9:**
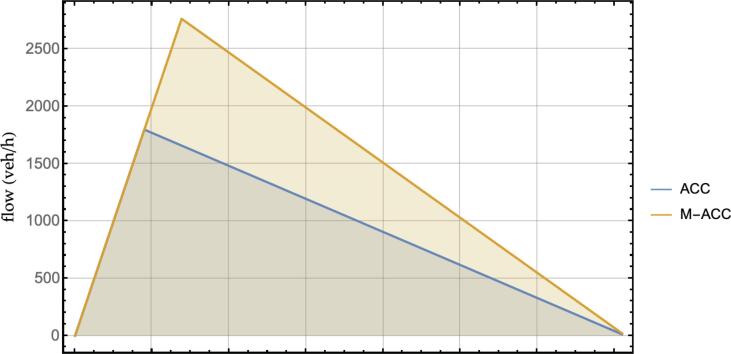
Fundamental diagram, ACC vs. M−ACC equivalent transfer function amplitude, $w_{L2}$ = 0.5, desired velocity = 100 km/h.

An example fundamental diagram where the time headway is reduced according to the equivalent transfer function criterion is showed in Fig. 9, for the ACC parameters in Table 1, desired speed 100 km/h and vehicle length l=5m. In order to draw Fig. 9, the traditional steady-state (constant velocity and equilibrium spacing) assumptions underlying the fundamental traffic flow diagram are introduced for the platoon.

### 4.2 M−ACC Flow generalization

**Fig. 10:**
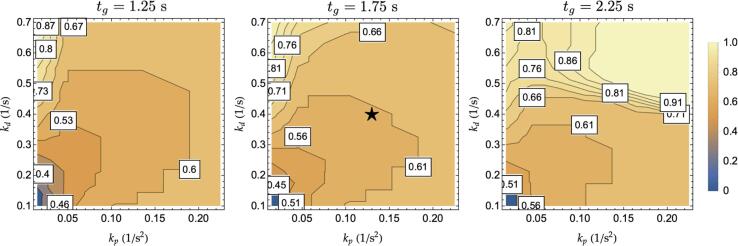
Time-gap scaling factors for a selection of controller gains, τ=0.1 s, T = 0.75 s.

Similarly to 3.3 M−ACC generalization, this paragraph investigates the impact of the M−ACC’s flow increase for a wide range of controller parameters. The study is carried out via computing the equivalent transfer function amplitude for the M−ACC in the $k_{p}-k_{d}$ plane and starting from a set of initial reference time-gaps. The results are graphically depicted in Fig. 10 for the same set of parameters as in Fig. 7. The amplitude of the charts in Fig. 10 encoded by means of the color is representative of the scaling factors multiplying the original headway, which yields the same transfer function maximum output. For example, the reference controller (star symbol in Fig. 10), which had an original $t_{g}=1.75s$, allows for a reduction of the time headway of 40% (corresponding to a scaling factor of 0.6 as in Fig. 10) upon the introduction of the multianticipation according to its position in the $k_{p}-k_{d}$ plane. Thus, the M−ACC transformation of the controller could be allowed to travel with a time-gap ≈1.05s while returning comparable string stability to the original ACC. From Fig. 10, poorly performing controllers, such as the ones having low gains and working at short time headway, benefit the most from the introduction of multianticipation. On the contrary, there is a minor additional advantage from applying multianticipation to controllers already close to string stability regions (≈0.9 multiplication factor for the top right controllers in the $t_{g}=2.25s$ case) given the chosen criterion to establish the equivalence.

The reduction in time-gap translates naturally into an increase in the maximum theoretical flow according to

(6) $$q_{\max}=\frac{V}{l+\eta +t_{g}V}.$$

**Fig. 11:**
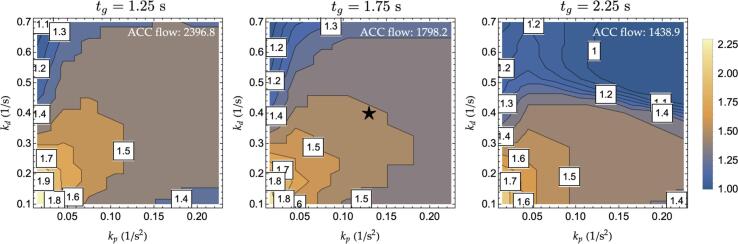
Maximum theoretical flow increase, multiplication factor over baseline ACC flow at 100 km/h.

Fig. 11 illustrates such a concept by depicting the multiplication factor that scales the baseline flow for a given headway (values in the top-right corner in *veh/h/lane*) at 100 km/h. For instance, considering the reference controller (star symbol in Fig. 11), it lies in a region that permits a multiplication factor of ≈1.5 over a baseline theoretical maximum flow of ≈1800 *veh/h/lane.* This translates into a theoretical flow from exploiting the multianticipation of ≈2700 *veh/h/lane* coherently with the fundamental diagram in Fig. 9.

Notice that any solution in between what has been discussed in the current paragraph (exploiting the full potential to maximize flow) and in Section 3.3 (exploiting the full potential to string stability) is also possible via the definition of an ad-hoc objective function. This paper further investigates the mentioned trade-off through Monte Carlo simulations in 5.2 Pareto optimization to establish the Pareto-optimality set.

## 5 Simulation investigation

Simulations have been carried out exploiting an internally developed micro-simulation framework to establish the real impact of multianticipation in a more realistic traffic scenario. The simulation environment allows, in fact, accounting for sources of non-linearity that the analytical modeling framework presented up to now cannot embrace. In particular, the maximum longitudinal acceleration the vehicles can exert is bounded in $\left[-8,2\right]m/s^{2}$ for both the ACC and M−ACC equipped vehicles, the RADARs’ detection range is limited to 250 *m*, the measurements are subjected to white noise, and a hybrid controller logic is implemented which replicates the switching between the car-following and free-flow behaviors depending on the lower demanded acceleration. The ACC and M−ACC−equipped virtual vehicles travel according to (2), (4) respectively, and the control laws are time-discretized and solved at 10 Hz to model the real-time implementation of the controllers. The simulation framework requires the user to input the leader’s velocity profile and the number of vehicles included in the platoon. Several databases are included in order to investigate the performance of the M−ACC over a wide range of perturbations. In particular:

• Selected velocity perturbations from the HighD dataset (Krajewski et al., 2018);
• In-house recorded data from the OpenACC database (Makridis et al., 2021).

**Fig. 12:**
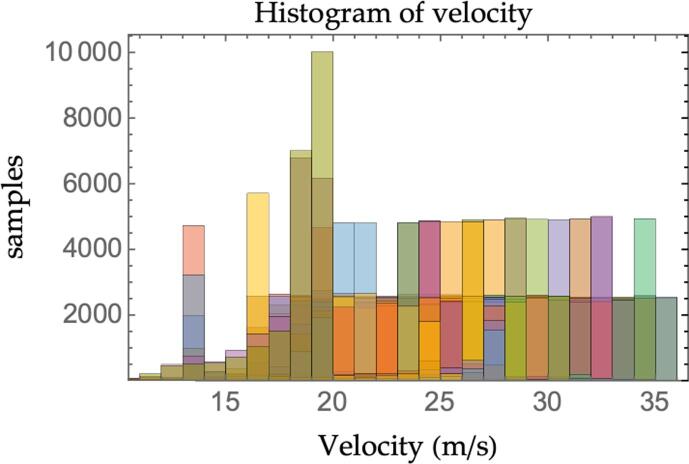
Simulation histogram of leader's velocity.

**Fig. 13:**
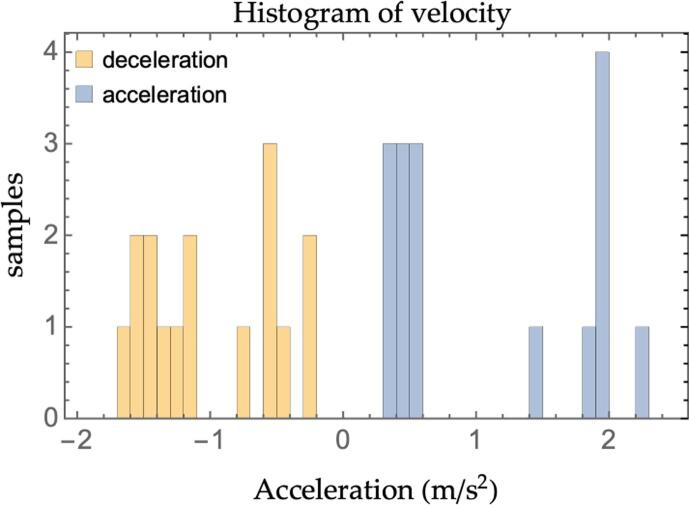
Simulation histogram of leader's perturbations.

The simulation setup is such that every vehicle in the platoon is in steady-state equilibrium status before the perturbation occurs. Selecting equilibrium conditions allows isolating the effect of the leader’s induced perturbation for the later computation of the string stability ratio. The practice is in line with state-of-the-art simulation-based research (Sun et al., 2018). Nonetheless, in the actual real-world functioning, perturbations might also originate from non-stationary conditions. The platoons are made up of 15 vehicles: 14 followers and one leader. The leader’s velocity profile is derived directly from the recorded trajectories in HighD and OpenACC datasets. In total, 16 trajectories are extracted from the databases: 9 real-world short perturbations from HighD and 7 experimental runs from the OpenACC dataset. Overall, the combination of the trajectories constitutes a 180 km, 2.75 h-long input for each simulation carried out. A statistical representation of the input dataset is proposed in Fig. 12 and Fig. 13. Fig. 12 depicts the leader’s velocities histogram at each simulation step for the whole dataset of perturbations (the total number of samples is thus the duration in time-steps of the whole dataset). Fig. 13, instead, reports the histogram of the minimum and maximum acceleration magnitude of the leader’s induced perturbations for each simulation (the total number of samples is thus two times the number of perturbations considered).

**Fig. 14:**
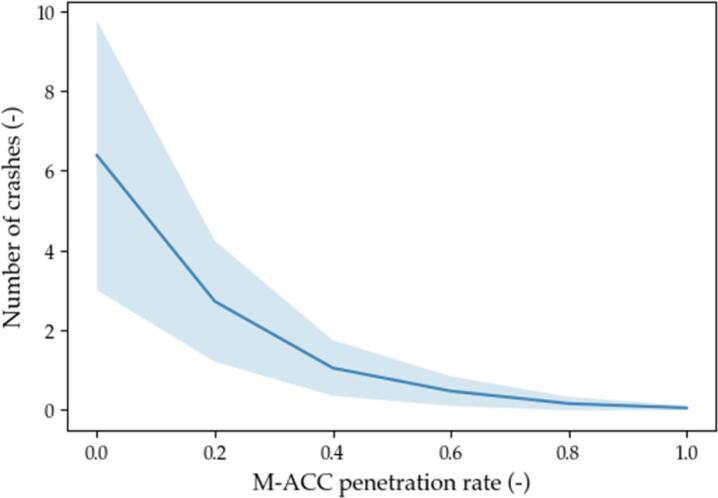
Number of crashes.

The charts show how the range of velocities considered is coherent with motorway/highway driving scenarios, a typical application for ACC. Similarly, the maximum/minimum values of the acceleration during the perturbations induced by the leader are quite mild and compatible with a smooth highway driving policy. We deliberately decided not to force hard decelerations to illustrate the degradation of the traffic flow and safety figures for the traditional ACC and to highlight the beneficial effect of multianticipation even for such moderate perturbations.

### 5.1 Stochastic simulations

The first usage of the simulation environment judges the macroscopic impact of M−ACC in a mixed flow environment where the followers can either be ACC (3) or M−ACC (4). The penetration rate of multianticipation is controlled by a simulation hyper-parameter which takes discrete values in [0, 0.2, 0.4, 0.6, 0.8, 1.0]. The set of parameters defining the car-following models are drawn from random uniform distributions to cover a wide range of variability of vehicle types. More specifically, the gains and delays in Table 2 stem from the experimental characterizations reported in (Gunter et al., 2021, Shi and Li, 2021, He et al., 2107) as mentioned in Section 2. The position of M−ACC cars within the platoon is subjected to stochasticity as well since the beneficial effect of the technology depends on the position of the M−ACC vehicles in the platoon (an M−ACC−based first follower will behave like an ACC whereas if the M−ACC were the last vehicle it would have limited capabilities of stabilizing the platoon). Notice, however, that due to the limited range of 250 *m* assumed for the RADAR model in the micro-simulation framework, the M−ACC may temporarily drive according to the ACC logic in case of very large headways. White noise is injected on RADAR’s model simulated measurements according to the accuracy metrics presented in (Hasch et al., 2012) for a 77 GHz operating frequency RADAR. The relative importance of the second leader was set to be 50% of the first leader ($w_{L2}$ = 0.5) coherently with the rest of the work. Additionally, given the lack of experimental datasets to support the reliability of the second leader distance and velocity detection, we assumed higher noise figures affecting $s_{2}$, $v_{L2}$ and a longer $T_{2}$ to partially account for the missing information. Nonetheless, a proper characterization of the measurements’ accuracy requires a real-world testing campaign which shall ascertain any discrepancy arising between the theoretical benchmark discussed in the current work and the actually achieved performance.

For each recorded trajectory and for each penetration rate, 100 sets of simulations are performed by assigning random parameters to the followers (values as in Table 2), and by defining random positions of the M−ACCs within the platoon. Ultimately a statistical analysis can be carried out over the 9600 simulations to establish the actual effect of multianticipation for a selection of KPIs.

**Fig. 15:**
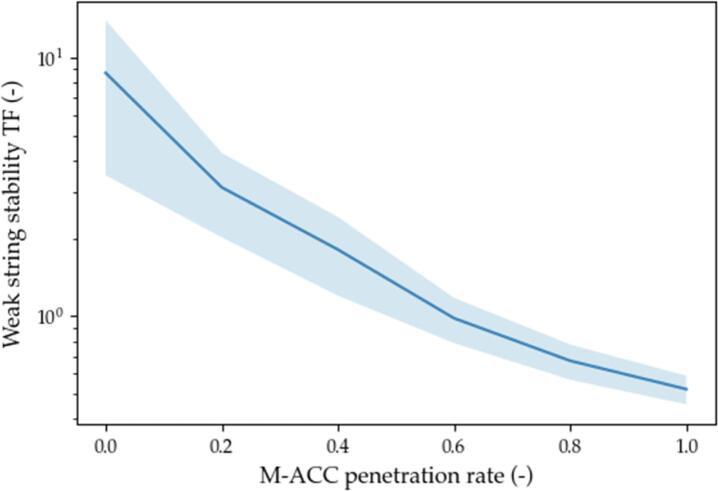
Weak string stability.

**Fig. 16:**
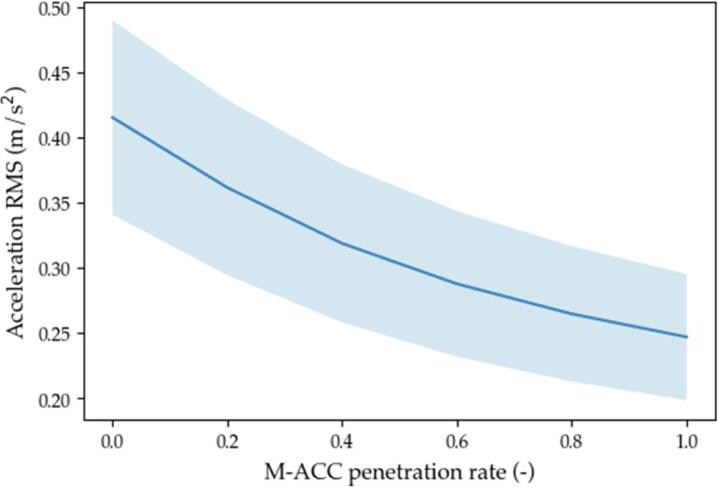
Acceleration RMS.

**Fig. 17:**
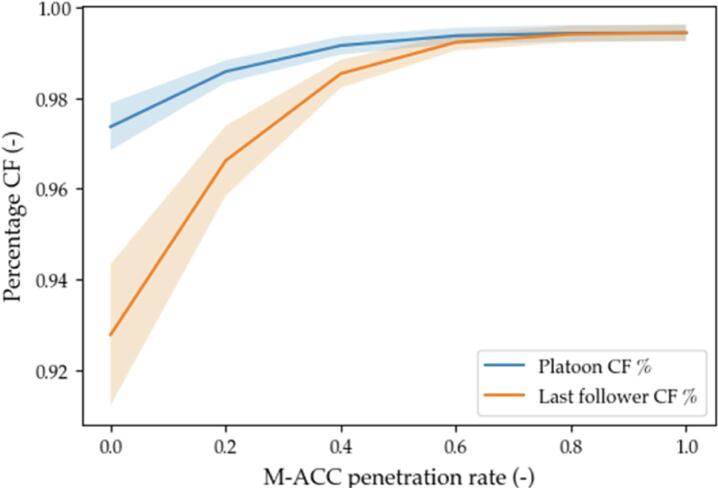
Percentage of time in car-follow.

**Fig. 18:**
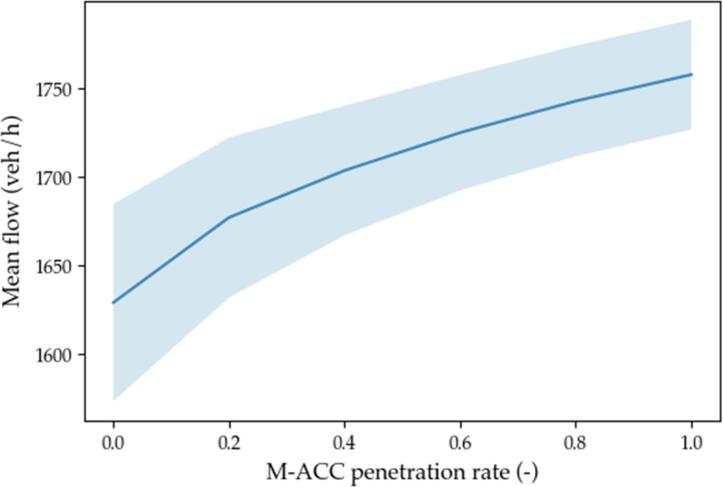
Mean traffic flow.

The metrics chosen to assess the effectiveness of the solution are representative of the performance of the M−ACC versus the traditional ACC on safety (number of crashes), on traffic throughput (weak string stability and percentage of time spent in car-follow), and on comfort (acceleration root means square value). The results are graphically represented in Fig. 15, Fig. 16, Fig. 17, Fig. 18 by means of the average value (solid thick line) and 1 standard deviation interval of confidence (light blue area).

#### 5.1.1 Results

Considering the “weak string stability” criterion in which string stability is defined by the maximum value of

(7) $$\|\frac{V_{\text{last\_follower}}\;\left(s\right)}{V_{\text{leader}}\;\left(s\right)}\|.$$

the beneficial effect of multianticipation scales proportionally to the penetration rate. In particular, a significant amount (≈60%) of M−ACC−equipped vehicles would be necessary in order to have a weak string stable platoon. That is due to the amplification of the dataset’s perturbations caused by the string instability which really pushes to the limit (and beyond) the tracking capabilities of the ACCs.

Concerning instead Fig. 14 where the thick line is the average value of crashes among the simulation sharing the same penetration rate, several crashes are reported when the penetration rate is below 20% due to the actual delay being comparable to the time-gap policy. In addition, a large variability of results characterizes low penetration rates. However, even a limited adoption of the M−ACC technology improves the platoon’s safety significantly. Given the minimalism of the modeling framework (no automatic emergency braking nor car-following logic disengagement are modeled), the obtained safety performance benchmark is only valid as a comparison between the two models (ACC vs. M−ACC) for the set of perturbations considered. Nonetheless, the decreased number of crashes for the M−ACC under the same headway settings suggests better capability for M−ACC to deal with traffic shockwaves.

The time spent in car-following in Fig. 17 is representative of the stability of the platoon (Makridis et al., 2020), as temporary free-flow transients might be experienced after a large undershoot in velocity due to strong deceleration. A limited introduction of the M−ACC provides a marginal advantage in terms of platoon stability (blue line in Fig. 17). However, since the overall platoon’s increase is mainly due to the last followers making up the platoon (orange line in Fig. 17), the actual small increase is nonetheless a more remarkable result.

Fig. 16 displays the RMS value of the longitudinal acceleration, which relates to comfort and fuel consumption. Even in this case, the M−ACC adoption returns noteworthy performance gains. Notice also that despite the M−ACC is capable of using higher instantaneous acceleration than the ACC (see Fig. 4), the superior string stability capabilities imply that the overall macroscopic figure in terms of RMS value over a long horizon is improved.

Macro-traffic metrics can be derived from the simulation data as well. More specifically, the traffic flow can be estimated as

(8) $$q=k\cdot {\overset{¯}{u}}_{s},$$

where k is the traffic density and ${\overset{¯}{u}}_{s}$ the harmonic mean of velocity. The increase in the average flow as from Fig. 18 is coherent with the results in Fig. 17 for the car-follow percentage.

**Fig. 19:**
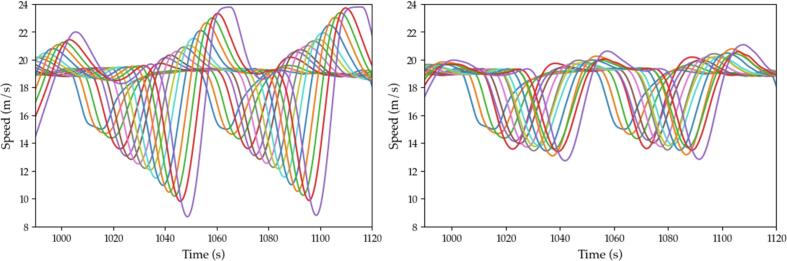
Velocity profiles, simulation scenario ACC vs. M−ACC 20% penetration.

Fig. 19 illustrates a close-up of the velocity profiles for a selected simulation for respectively the ACC case (left) and the 20% penetration rate of M−ACC (right). A contained penetration of the multianticipation yields visible improvement despite not annihilating the string instability phenomenon.

**Fig. 20:**
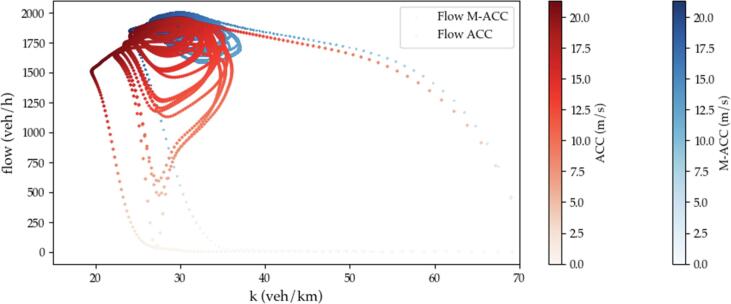
Fundamental diagram ACC vs. M−ACC 20% penetration.

Finally, the chart in Fig. 20 shows the resultant fundamental diagram for the same simulation scenario as in Fig. 19 for both the ACC case (red dots) and the M−ACC solution (blue dots). The chart is obtained via computing the instantaneous flow at each simulation step using the vehicles velocities harmonic mean as in (8). Fig. 20 demonstrates how the equilibrium flow for the M−ACC case stabilizes around a higher peak and a higher density area. In addition, it is particularly evident how the perturbations do not translate into noticeable flow drops for the multianticipation case.

### 5.2 Pareto optimization

The simulation framework can finally be exploited to establish the Pareto optimality conditions for both the ACC and the M−ACC cases. The problem of tuning the time-gap for a car-following application, from a technological perspective, is indeed a trade-off solution between maximizing flow and minimizing string instability. Such a trade-off resolves in a multi-objective optimization which ultimately originates the Pareto frontier for the given cost functionals. Exploiting Pareto-like optimization methods for the synthesis of an optimal controller is not a novelty. For instance, in (Mba and Novara, 2016), a similar framework is proposed to compute the Pareto frontier of ACC controllers given the acceleration vs. velocity tracking capability trade-off. However, the analysis does not account for user-specific headway preferences as, in principle, a driver is free to select the headway policy provided by the featured ACC/M−ACC and might not be willing to use a technology that does not make him/her feel comfortable. As such, the human preference is out of the scope of the optimization procedures given the unpredictability and sparsity of human drivers’ headway.

The proposed optimization stems from a Monte Carlo workflow: for the selection of scenarios in the previous section, 50 simulations are performed for a given time-gap while the rest of the parameters are left subjected to stochasticity, as from Table 2. The procedure is repeated for the case where all the vehicles making up the platoon are ACC-based and for a 20% penetration rate of the M−ACC. Ultimately, the introduction of M−ACC capable vehicles should result in the *Pareto frontier* shifting towards a set of optimization parameters that would dominate the ACC Pareto-optimality set. The metrics selected to reproduce the mentioned trade-off truthfully are the average flow (8) and the weak string stability criterion (7). In order to have both objective functions to be maximized, the weak string stability metric is here computed as the reciprocal of (7).

**Fig. 21:**
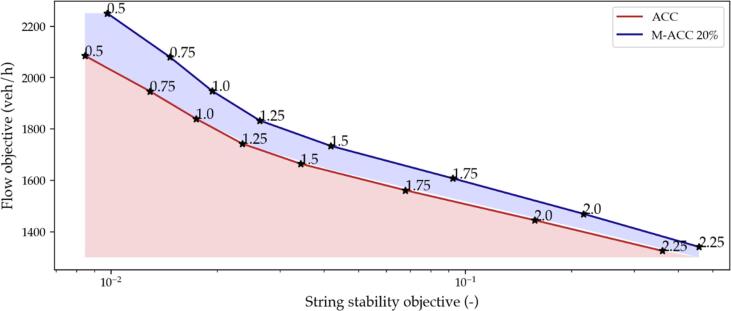
Pareto frontier ACC vs. M−ACC 20% penetration.

The results are graphically depicted in Fig. 21, where the identified frontiers by means of the Monte Carlo method are represented. The light red area is the dominated region for ACC controllers, whereas the light blue area is the region dominated by the M−ACC, which is not attainable for the ACC controllers. The annotated points on the frontiers are the values for the time-gap policy adopted in seconds. As expected, the M−ACC yields a noticeable shift towards the top-right corner (the direction of the global optimum) of the Pareto frontier. The identified frontier enables the design of controllers better optimizing both the string stability and flow objectives.

## 6 Conclusions

A worrisome trade-off between traffic flow and string stability, for current commercial ACCs, has been recognized in recent literature. This could have detrimental effects on future motorway traffic. One of the main solutions proposed in the literature is the use of connectivity to increase traffic flow and stabilize vehicle platoons. However, connectivity may have to overcome a number of obstacles before becoming widely available in the market. As an alternative solution to CACC-based technology, the paper has proposed the adoption of multianticipation in the car-following task. The work is motivated by the well-known advantages of multianticipation and the recent advancements in exploiting RADARs’ generated evidence. The scientific effort encompasses both the expected theoretical benefits through an in-depth study of the analytical properties of the closed-loop system and a simulation-based validation of the control logic in a more realistic application domain. Indeed, from the presented results, even a “limited” multianticipation which only accounts for a second leader would guarantee increased safety, flow, and string stability for the traffic flow. An extensive study was carried out to ensure the correct functioning of the logic over a wide range of calibrated parameters. Eventually, the Pareto frontiers assessment utilizing Monte Carlo simulations resolves the generalization capability of the control logic by showing a clear shift of the Pareto set towards solutions better optimizing at the same time flow and traffic stability.

Further work will be devoted to the setup of an adaptive headway policy which possibly exploits the two leader velocities to infer the instantaneous traffic stability conditions and to adapt to the disturbances consequently (e.g., increasing the headway when the two leaders’ velocities are distant and reducing the headway for minor discrepancies in order to maximize flow). Besides, the tuning of the parameter $w_{L2}$ was not discussed but instead assigned with a conservative approach based on heuristic considerations. A more realistic tuning procedure might be required via exploiting a Pareto-like optimization where string stability performance vs. noise robustness appears in a trade-off fashion following characterization of the real-world detection capability of the recently introduced technology. Eventually, a comparison involving our proposed M−ACC vs*.* a state-of-the-art CACC solution would return the complete picture of the M−ACC’s effectiveness.

### CRediT authorship contribution statement

**Riccardo Donà:** Conceptualization, Methodology, Formal analysis, Data curation, Visualization, Software, Writing – original draft, Writing – review & editing. **Kostantinos Mattas:** Conceptualization, Methodology, Writing – original draft. **Yinglong He:** Data curation, Software. **Giovanni Albano:** Data curation. **Biagio Ciuffo:** Conceptualization, Writing – review & editing, Funding acquisition.

## Declaration of Competing Interest

The authors declare that they have no known competing financial interests or personal relationships that could have appeared to influence the work reported in this paper.

## Footnotes

1 The same manufacturer is transitioning to a cameras-only sensor setup in the US market. Thus, upcoming vehicles might not feature multianticipation.
